# Supplementary material for: C-terminal eYFP fusion impairs Escherichia coli MinE function
Source: Open Biol. 2020 May 27;10(5):200010. doi: 10.1098/rsob.200010 (PMC7276532; doi:10.1098/rsob.200010)
Supplement: Supplemental material [file rsob200010supp1.pdf]

C-terminal eYFP fusion impairs *Escherichia coli* MinE function

Navaneethan Palanisamy, Mehmet Ali Öztürk, Emir Bora Akmeriç and Barbara Di Ventura

Supplemental Material

Figures S1-5

References

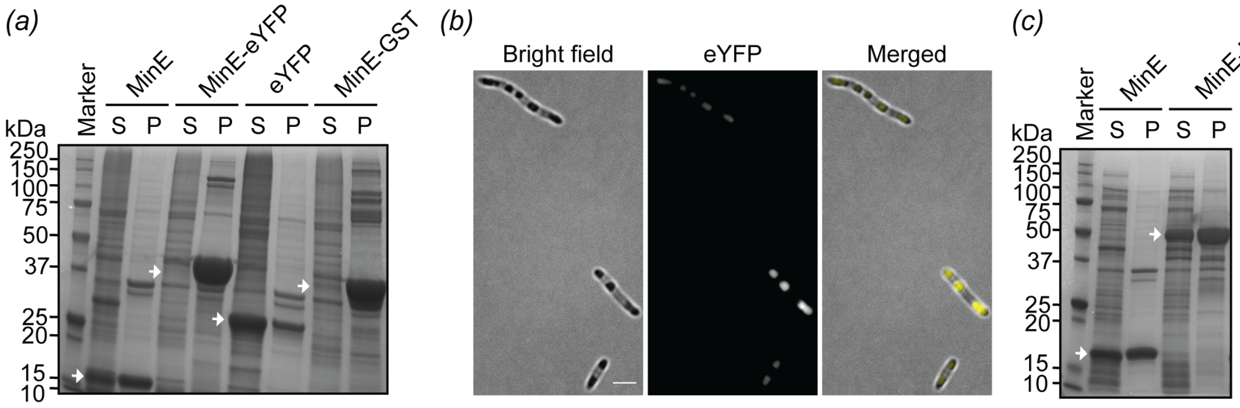

Figure S1. MinE-eYFP forms aggregates *in vivo* at high concentrations and is found in the insoluble fraction. (*a*) and (*c*) SDS-PAGE analysis of the soluble and insoluble fractions of *E. coli* Rosetta™ (DE3) pLysS cells transformed with the indicated constructs. The white arrowhead indicates the band representing the overexpressed protein. For simplicity, the arrowhead is shown only next to the soluble fraction. (*b*) Representative fluorescence microscopy image of *E. coli* Rosetta™ (DE3) pLysS cells transformed with pET28a-MinE-eYFP induced for 3 hours with 1 mM IPTG at 37 °C.

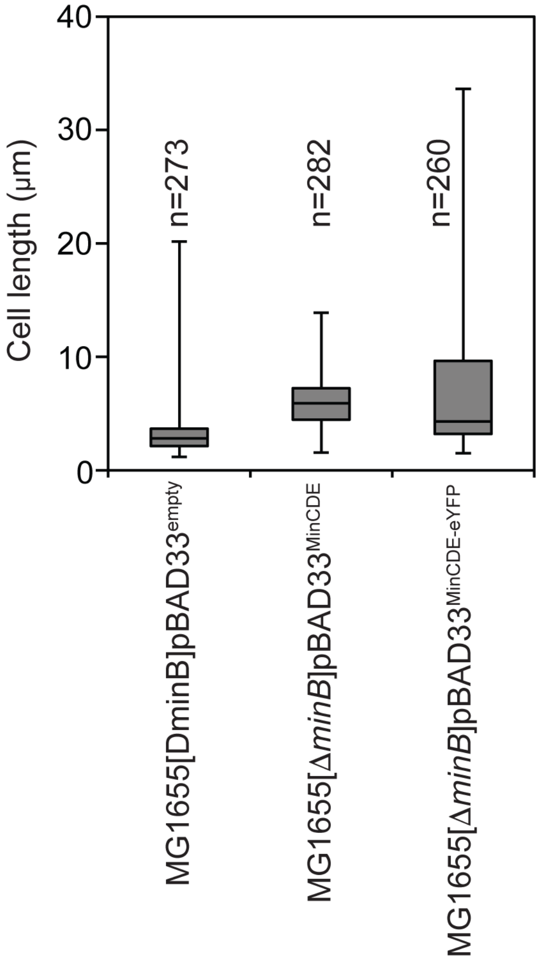

Figure S2. Expression of MinCDE-eYFP with 0.1% arabinose leads to cell elongation. Box plot showing the cell length of MG1655Δ*minB* cells transformed with the indicated plasmid and induced with 0.1% arabinose. n, total number of cells quantified.

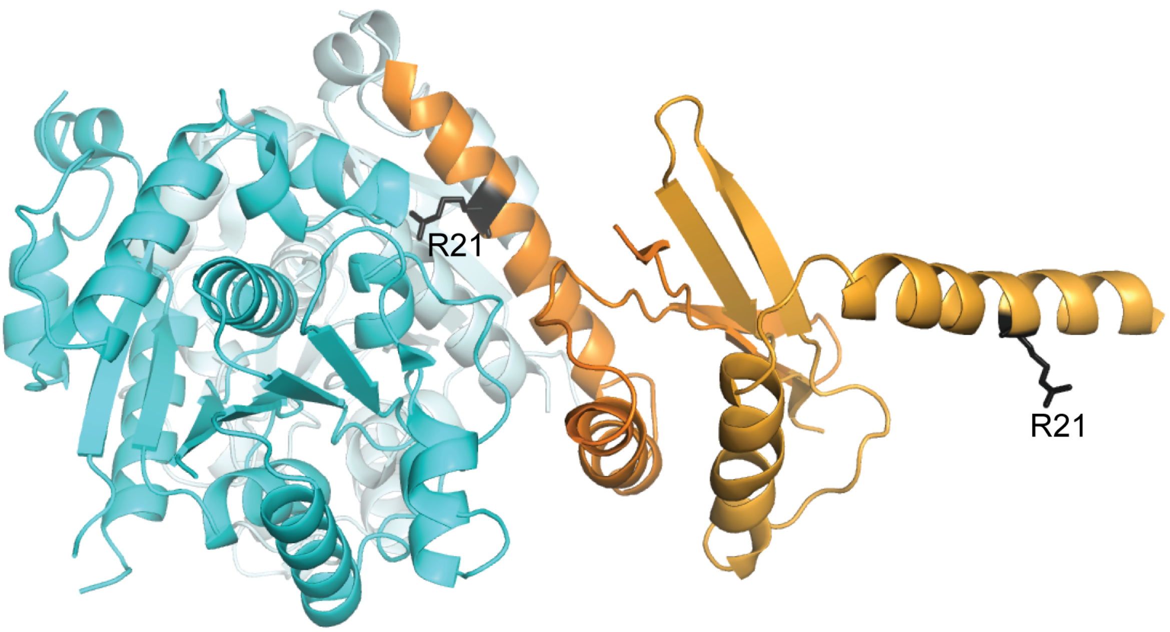

Figure S3. X-ray crystal structure of *E. coli* MinD in complex with MinE. Cartoon representation of the structure of *E. coli* MinD dimer (blue - pale blue) bound to *E. coli* MinE dimer (orange - dark orange) (PDB id: 3R9J). Arginine at position 21 (R21), involved in triggering MinD ATPase activity, is highlighted in black.

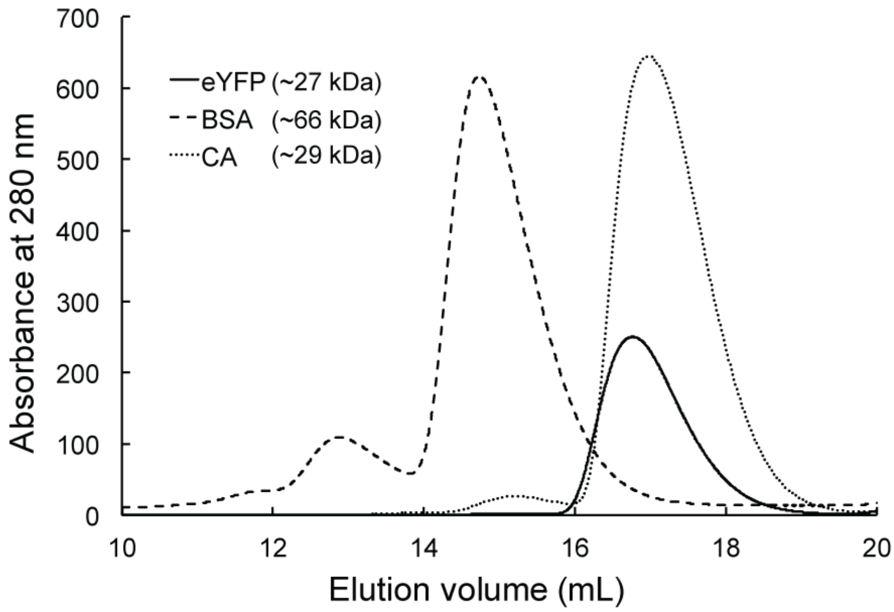

Figure S4. eYFP elutes as a monomer. Size-exclusion chromatography of eYFP (13 µM). As molecular mass standards we used bovine serum albumin (BSA; 15 µM) and carbonic anhydrase (CA; 15 µM). eYFP has the A206K mutation, known to favor the monomeric state of the protein [1, 2].

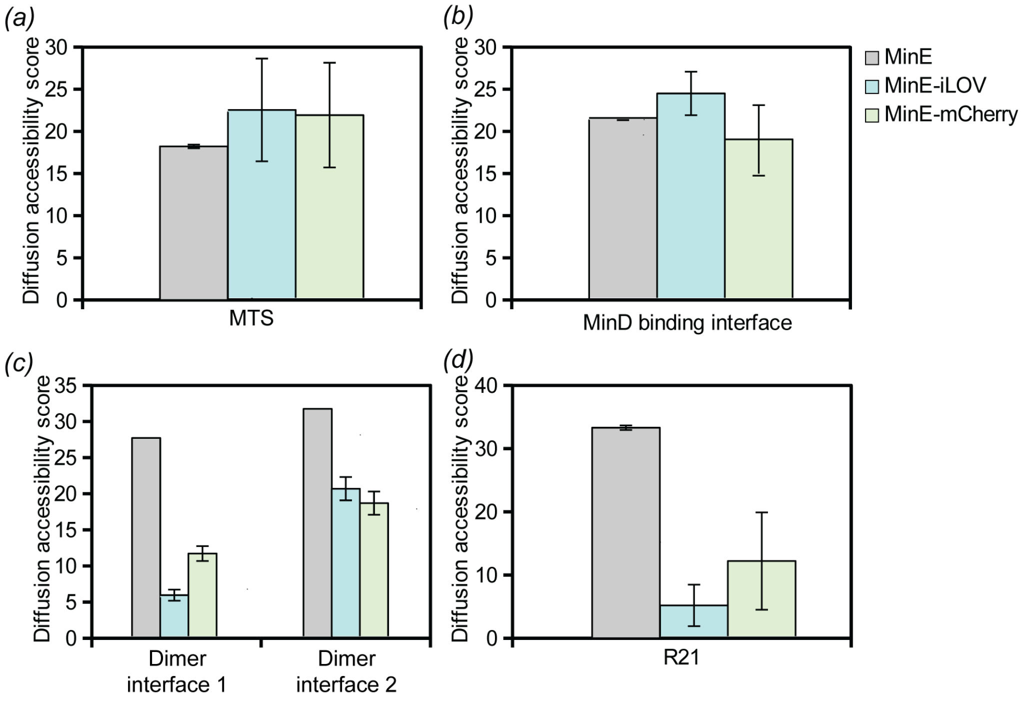

Figure S5. Computational analysis of MinE-mCherry and MinE-iLOV fusion proteins. Bar plots showing the diffusion accessibility scores for the indicated functional elements. Values represent the mean and error bars represent the standard deviation of three independent simulations.

References

1. Kentner D, Sourjik V. Dynamic map of protein interactions in the Escherichia coli chemotaxis pathway. Molecular Systems Biology. 2009;5. doi: ARTN 238 10.1038/msb.2008.77. PubMed PMID: WOS:000263167600004.

2. Zacharias DA, Violin JD, Newton AC, Tsien RY. Partitioning of lipid-modified monomeric GFPs into membrane microdomains of live cells. Science. 2002;296(5569):913-6. doi: DOI 10.1126/science.1068539. PubMed PMID: WOS:000175442500045.
